# circMMD reduction following tumor treating fields inhibits glioblastoma progression through FUBP1/FIR/DVL1 and miR-15b-5p/FZD6 signaling

**DOI:** 10.1186/s13046-023-02642-z

**Published:** 2023-03-17

**Authors:** Shengchao Xu, Chengke Luo, Dikang Chen, Lu Tang, Quan Cheng, Ling Chen, Zhixiong Liu

**Affiliations:** 1 Department of Neurosurgery, Xiangya Hospital, Central South University, No.87, Xiangya Road, Changsha, 410008 Hunan China; 2 National Clinical Research Center for Geriatric Disorders, Xiangya Hospital, Central South University, Changsha, 410008 China; 3 Hunan An Tai Kang Cheng Biotechnology Co., Ltd, Changsha, 410008 China; 4 Department of Anesthesiology, Xiangya Hospital, Central South University, ChangshaHunan, 410008 China; 5 Department of Neurosurgery, Chinese People’s Liberation Army of China (PLA) General Hospital, Medical School of Chinese PLA, Institute of Neurosurgery of Chinese PLA, Beijing, 100853 China

**Keywords:** Tumor treating fields, Glioblastoma, circRNA, Wnt/β-catenin signaling

## Abstract

**Background:**

Tumor treating fields (TTF) is the latest treatment for GBM. Circular RNA (circRNA) has been demonstrated to play critical roles in tumorigenesis. However, the molecular mechanism of TTF remained largely unknown and the role of circRNA in TTF was not reported. The aim of this study was to elucidate the role and mechanism of circMMD in TTF treatment of GBM.

**Methods:**

Divergent primer was designed to verify the existence of circMMD in GBM cells. The prognostic role of circMMD was explored in glioma specimens. The knockdown and overexpressed plasmids were used to evaluate the effect of circMMD on GBM cell proliferation and TTF efficacy. RNA pull-down and RNA immunoprecipitation were performed to identify binding proteins of circMMD. Subcutaneous and intracranial tumor models were established to validate findings in vivo.

**Results:**

The expression of circMMD was elevated in GBM and its high expression indicated poor prognoses. TTF intervention could reduce circMMD synthesis, which suppressed GBM proliferation and increased TTF-mediated apoptosis. The reduction of circMMD promoted the interaction between FUBP1 and FIR, which decreased DVL1 transcription. Meanwhile, decreased circMMD would promote the activity of miR-15b-5p to degrade FZD6. Finally, the diminished expression of DVL1 and FZD6 expression suppressed the activation of Wnt/β-catenin pathway.

**Conclusions:**

Our study revealed a novel mechanism of TTF that TTF-mediated reduction of circMMD could inhibit Wnt/β-catenin pathway to suppress GBM proliferation.

**Supplementary Information:**

The online version contains supplementary material available at 10.1186/s13046-023-02642-z.

## Background

Glioblastoma (GBM) is one of the most malignant and aggressive cancers in the central nervous system [1]. The median overall survival (OS) of GBM patients is 14.4 months and the 5-year survival rate is less than 5% [2]. Current treatments for GBM include surgery, chemotherapy and radiotherapy. However, tumor recurs in most GBM cases, and few therapies can be used for recurrent GBM. Tumor treating fields (TTF) are a kind of physical therapy that exerts alternating low-intensity, intermediate-frequency electric fields to inhibit cancer proliferation [3]. Its efficacy has been established by a large-scale clinical trial [4], which confers its approval for both newly diagnosed and recurrent GBM. However, its molecular mechanism remains largely obscure.

Circular RNA (circRNA) is a kind of non-coding RNAs that plays a critical role in regulating cancer progression [5]. The production of circRNA mainly relies on the direct back-splicing or exon skipping of precursor mRNA [6]. Some RNA-biding proteins (RBPs) such as eukaryotic translation initiation factor 4A3 (EIF4A3) and FUS RNA binding protein (FUS) could bind to the flank region of circRNA to promote its biogenesis [7, 8]. Due to its unique structure, circRNA has four main regulatory mechanisms: [1] sponge micro RNAs (miRNAs) to inhibit miRNA activity and regulate the expression of target genes [9]; (2) regulate linear splicing of precursor RNA [10]; (3) act as a proteins scaffold to regulate protein function [11]; (4) translate small peptide that exerts regulatory activities [12]. For example, Zhu et al. revealed that circMMD_007 (hsa_circ_0002015) could sponge miR-197-3p to elevate PTPN9 expression so as to promote lung adenocarcinoma progression [13]. Moreover, circ-E-cad could encode a small peptide to activate EGFR-STAT3 signaling to promote GBM progression [12]. And circACTN4 was shown to facilitate intrahepatic cholangiocarcinoma progression via binding with YBX1 to promote FZD7 transcription [14]. However, no study has revealed the role of circRNA in TTF treatment of GBM.

In this study, we used TTF system (CL-301A) that was self-developed by our research team. Its efficacy to inhibit GBM progression has been demonstrated both in vitro and in vivo [15–17]. Our study would provide novel insights into the therapeutic mechanism of TTF against GBM.

## Materials and methods

### Cell culture and TTF intervention

DBTRG-05MG (DBTRG) and U251 cells were used in this study and were obtained from ATCC (USA) and Procell (China), respectively. The TTF device for cell culture was provided by Antai Kangcheng Biotechnology Co., Ltd. TTF intervention was applied as previously described with a frequency of 200 kHz and an intensity of 1.5 V/cm [17].

## Public sequencing data extraction

RNA-seq data with corresponding clinical information were extracted from The Cancer Genome Atlas (TCGA), Chinese Glioma Genome Atlas (CGGA), and Gene Expression Omnibus (GEO) databases as previously described [18–20]. In this study, TCGA-lower-grade glioma (LGG) and TCGA-GBM datasets were extracted and combined into TCGA-gliomas dataset. CGGA-325 and CGGA-693 were combined into CGGA-gliomas dataset. The batch effect was removed by “sva” R package. The other three microarray datasets (CGGA-301, GSE108474, and GSE16011) of gliomas were also extracted. LGG was defined as grade II and III gliomas relative to GBM (grade IV). Chromatin immunoprecipitation (ChIP)-seq data of epigenetic modification in GBM were extracted from GSE121719 [21]. Integrative Genomics Viewer (IGV) software was used for visualization.

### Cell viability and 5-Ethynyl-2’-Deoxyuridine (EdU) assays

Cell viability and EdU assays were conducted as previously reported [17]. Cell viability was estimated using Cell Counting Kit CCK-8 (Dojindo, Japan) and EdU assay was performed using EdU assay kit (Ribobio, China). Cells were stained with Apollo 567 and DAPI and detected using Eclipse Ti2-A fluorescence microscope (Nikon, Japan).

### Tumor mice model and immunohistochemistry (IHC)

All animal experiments were approved by the Ethics Committee of Xiangya Hospital (No. 202103148) following the Guide for the Care and Use of Laboratory Animals. To establish subcutaneous tumor model, 1 × 10^6^ DBTRG cells were injected at the flank of 6-week nude mice (*n* = 5 per group). Tumor volume was determined using the following equation: length (L) × width2 (W) × 0.5. Orthotopic mice model was established as previously described [22]. A total of 1 × 10^5^ DBTRG cells were injected into mice brain with a rate of 1 μL/min. Mice were sacrificed according to the following criteria: weight loss of 20–25%; tumor weight reached 10% of mice weight; appetite loss of more than 24 h; depression and hypothermia. After sacrifice, mice brain and subcutaneous xenograft were subjected to hematoxylin–eosin (HE) staining and IHC, respectively. IHC was conducted as previously reported [19]. The specimens were embedded in paraffin. The 0.3% H_2_O_2_ and 5% BSA were used for the blockade. The primary antibodies were applied at 4 °C overnight. After the application of secondary antibody, diaminobenzidine tetrahydrochloride (DAB) and hematoxylin were used for staining.

### Quantitative real-time PCR (qRT-PCR)

Total RNA was extracted by TRIzol Reagent (Invitrogen, USA). RNA of nucleus and cytoplasm was extracted using PARIS Kit (Invitrogen, USA). Then the cDNA was synthesized using PrimeScript RT reagent Kit (Takara, RR047A). The qRT-PCR was conducted on QuantStudio 5 Real-Time PCR System (ABI, Thermo). GAPDH and U6 were used as the internal control for mRNA and miRNA expressions, respectively. The primers of hsa-miR-15b-5p and U6 were purchased from Ribobio Co. Ltd (Guangzhou, China) and others were listed in Table S1.

### RNA immunoprecipitation (RIP)-qPCR

The RIP assay was conducted using RNA Immunoprecipitation kit (Geneseed, China) following the instruction. Briefly, cells were lysed and incubated with anti-flag, anti-FUBP1, or anti-EIF4A3 antibodies. Protein A/G Magnetic Beads were used to collect RNA–protein complex. After RNA extraction and purification, it was subjected to qRT-PCR as indicated.

### ChIP-qPCR and ChIP-seq

The ChIP assay was conducted using Pierce Magnetic ChIP kit (Thermo) following the instruction. In brief, cell nuclei were collected and sonicated using Bioruptor with 30 s-ON/ 30 s-OFF for 20 cycles. Then 5 μg anti-flag, anti-FUBP1, or anti-FIR antibodies were incubated in sheared chromatin overnight. DNA was extracted by ChIP Grade Protein A/G Magnetic Beads and eluted by Elution Buffer. After DNA purification, it was subjected to qPCR or ChIP-seq. ChIP-seq was conducted by Novegene (Beijing, China).

### RNA pull-down, silver staining and mass spectrometry analysis

The RNA pull-down assay was conducted using Pierce Magnetic RNA–Protein Pull-Down Kit (Thermo, USA) following the instruction. The biotin-labeled probes targeting the junction site of circMMD were synthesized by RiboBio (Guangzhou, China). Cell lysates were incubated with the probe and the extracted protein was subjected to Western blot. Silver staining was conducted using the Fast Silver Staining Kit (Beyotime, China) following the protocol. The mass spectrometry analysis was performed by Shanghai Applied Protein Technology (Shanghai, China).

### RNase R and Actinomycin D treatment

To verify the stability of circMMD, 10 μg RNA extracted from DBTRG and U251 cells was divided into two parts: one was incubated with 1 μl RNase R (20 U/μl, Geneseed, China) at 37 °C for 30 min; another one was treated without RNase R. The RNA was incubated at 70 °C for 10 min to inactivate RNase R and then subjected to qRT-PCR. Moreover, cells were treated with 2 μg/ml Actinomycin D (Sigma, USA) for 0, 8, 16, and 24 h. Then their RNAs were extracted and subjected to qRT-PCR.

### Dual-luciferase reporter assay

The predicted binding sequence of circMMD and hsa-miR-15b-5p or hsa-miR-424-5p was cloned into pGL3-basic vector. Also, the -2000 ~ -1 and -1422 ~ -1261 downstream sequences of DLV1 and mutant sequence were cloned into pGL3-promoter vector (General Bio, China). TOP/FOP flash plasmids were synthesized using sequences from Addgene. For dual-luciferase assay, the plasmids were transfected into 293 T cells using Lipofectamine 3000. After 48 h, luciferase activity was measured using Dual-Luciferase Reporter Assay System (Promeaga, USA) and renilla luciferase activity was used to scale the transfection efficiency.

### Clinical samples and fluorescence in situ hybridization (FISH)

Human glioma microarray containing 82 glioma tissues and 5 normal brain tissues was obtained from Servicebio (Wuhan, China), and clinical information was provided in Table S2. The experiment was approved by Ethics Committee of Xiangya Hospital (No. 202103148). To quantify circMMD expression, the glioma microarray was hybridized with Cy3-labeled circMMD probe RiboBio (Guangzhou, China) as listed in Table S1. The circMMD expression was calculated based on the percentage of positive cells. FISH in DBTRG and U251 cells was conducted as previously reported [17]. Cells were fixed with 4% paraformaldehyde and treated with PBS containing 0.5% Triton X-100. Then cells were incubated with probes at 37 °C overnight. After three times of washing, cells were stained with DAPI. Then the fluorescence was detected using Nikon Eclipse Ni-U microscope or Nikon Eclipse C2 confocal microscope.

### Flow cytometry

Cell apoptosis was evaluated as previously reported [17]. The Annexin V-APC and PI was used to detect cell apoptosis.

### Immunofluorescence

Cells were fixed with 4% paraformaldehyde for 15 min at room temperature. Then 0.5% Triton X-100 PBS was added for 10 min. The primary antibodies targeting FUBP1 or FIR were applied and the FITC- or Cy3-labeled anti-rabbit or anti-mouse antibody was applied. Cell nuclei were stained by DAPI. The fluorescence was detected as indicated.

### RNA sequencing and enrichment analyses

RNA sequencing of sh-circMMD-1 and sh-NC DBTRG cells was conducted by Novogene Bioinformatics Technology Co., Ltd (Beijing, China). Enrichment analysis was conducted using “clusterprofiler” R package including Gene Ontology (GO) and Kyoto Encyclopedia of Genes and Genomes (KEGG), in which GO contained three items: biological process (BP), cellular component (CC) and molecular function (MF). Those with false discovery rate (FDR) ≤ 0.05 were selected.

### Co-immunoprecipitation (Co-IP) and Western blot

Cell lysate was prepared using RIPA lysis buffer (Beyotime, China) with Protease inhibitor Cocktail (MCE, China). The 5 μg anti-flag, anti-FUBP1, or anti-FIR antibodies were added to 1 mg total protein at 4 ℃ overnight. Then 50 μl Protein A/G Magnetic Beads (MCE, China) was added and incubated for 2 h. After washes, 50 μl SDS loading buffer was added to suspend the beads. The protein was separated in 10% SDS-PAGE gel and transferred to the PVDF membrane (Millipore, USA). After the blockade with 5% skim milk for 1 h at room temperature, the membrane was incubated with primary antibodies at 4 °C overnight. The secondary antibody HRP-conjugated anti-rabbit or anti-mouse IgG was applied for 1 h at room temperature and the protein content was detected using an enhanced chemiluminescence system. The primary antibodies used in this study was listed in Table S1.

### RNA interference, plasmid construction and lentivirus production

The siRNAs were obtained from GenePharma (Jiangsu, China). Vectors containing shRNA targeting circMMD and overexpressed circMMD plasmids were obtained from GeneChem (Shanghai, China). The pLVX-puro, pLKO.1, and pcDNA3.1 vectors were obtained from General Bio (Anhui, China). The sequence of siRNAs and shRNAs were listed in Table S1. The Lipofectamine 3000 was used for cell transfection. To produce lentivirus, lentiviral vectors were co-transfected with psPAX2 and pMD2.G (Addgene) for 48 h. Then supernatant containing lentivirus was collected.

### Statistical analysis

Data analyses and visualization were conducted using GraphPad Prism 8 and R 3.6.3. The difference comparison was performed using student’s t-test or one-way ANOVA between two or more than two groups. The *p*-value < 0.05 was considered statistically significant.

## Results

### circMMD expression was decreased after TTF intervention and correlated with glioma prognosis

**Fig. 1:**
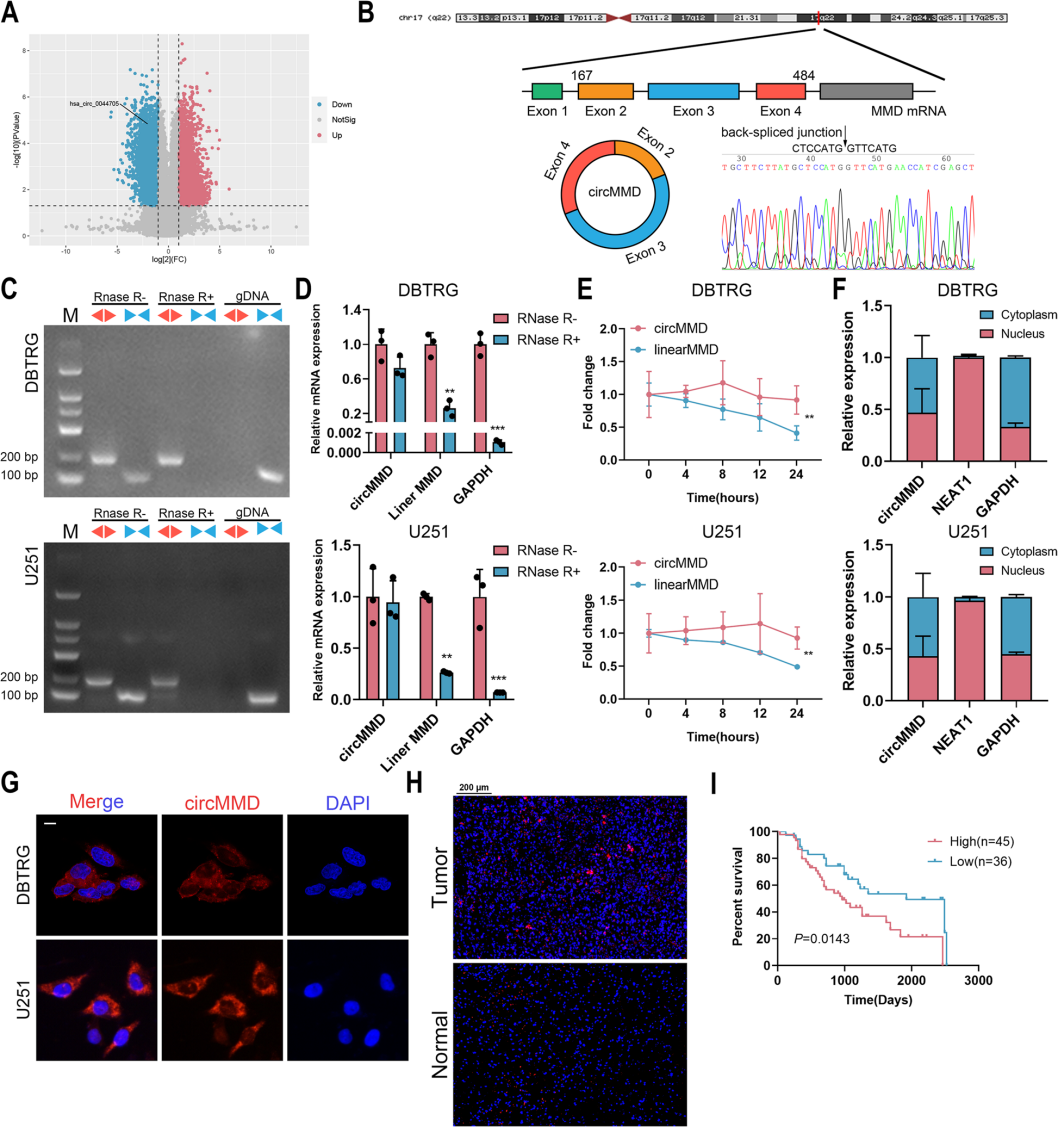
Identification of circMMD and its prognostic value in GBM. **A** Volcano plot of differentially expressed circRNAs between TTF and control groups and circMMD was identified. **B** Sanger sequencing of the back-spliced junction of circMMD. **C** Amplification of circMMD and linear MMD from cDNA or gDNA using divergent (red arrow) or convergent primers (blue arrow). **D** Relative expression of circMMD, linear MMD, and GAPDH after RNase R treatment or not. **E** The abundances of circMMD and linear MMD in DBTRG and U251 cells treated with actinomycin D at the indicated time points. **F** Relative abundance of circMMD in nucleus and cytoplasm of DBTRG and U251 cells. **G** FISH assay detected the localization of circMMD in DBTRG and U251 cells. Scale bar = 10 μm. **H** FISH assay detect the abundance of circMMD in GBM tumor specimen and normal brain tissue. Scale bar = 200 μm. **I** Kaplan–Meier analysis of correlation of circMMD expression with overall survival of glioma patients. ** *P* < 0.01, *** *P* < 0.001, # *P* > 0.05

To explore the role of circRNAs in TTF treatment, we conducted whole transcriptome sequencing as previously described [17], which identified 13,903 differentially expressed circRNAs including 8,508 upregulated and 5,395 downregulated ones. Among downregulated circRNAs, circMMD (hsa_circ_0044705) was selected for further analysis (Fig. 1A). The 318-nt circMMD was formed by circularization of exon 2–4 of *MMD* gene. The back-spliced junction of circMMD was confirmed by Sanger sequencing, which was consistent with the circBase database (Fig. 1B). In contrast to linear MMD transcribed by convergent primers, circMMD was resistant to the digestion by RNase R and could not be amplified from gDNA (Fig. 1C). Similarly, linear MMD and GAPDH were degraded by RNase R whereas circMMD was not (Fig. 1D). To determine RNA stability, ActD was applied to stop gene transcription, and compared with linear form, circMMD had a relatively high stability (Fig. 1E). Further, we found that circMMD distributed in both the nucleus and cytoplasm of DBTRG and U251 cells (Fig. 1F, G). Compared with normal brain tissue, circMMD was highly expressed in glioma, and its high expression indicated poor prognoses (Fig. 1H, I). Also, circMMD expression was significantly correlated with tumor grade and IDH1 status in gliomas (Table S3). These findings suggested that cicMMD was a prognostic biomarker for glioma patients and might be a target of TTF to inhibit glioma progression.

### circMMD promoted GBM proliferation both in vitro and in vivo

**Fig. 2:**
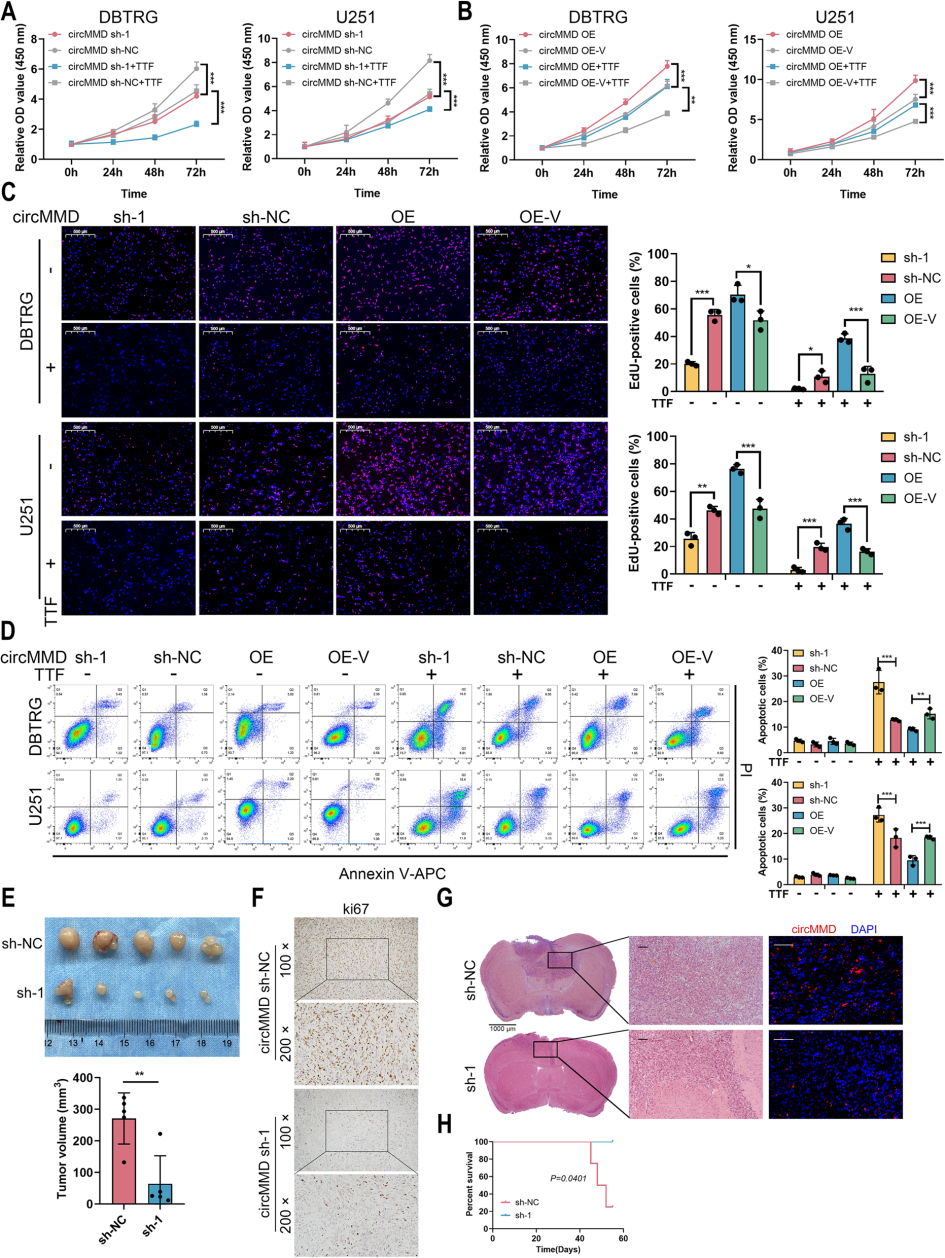
Knockdown of circMMD inhibited GBM proliferation and promoted TTF-mediated apoptosis. **A-B** CCK-8 assay of DBTRG and U251 cells after knockdown (**A**) or overexpression (**B**) of circMMD in the presence or absence of TTF intervention for 48 h. **C-D** EdU assay (**C**) and flow cytometry (**D**) of DBTRG and U251 cells after knockdown or overexpression of circMMD in the presence or absence of TTF intervention for 48 h. Scale bar = 500 μm. **E** Tumor volume of subcutaneous xenograft of sh-circMMD-1 or sh-NC DBTRG cells. **F** Ki67 staining of subcutaneous xenograft. **G-H** Intracranial tumor model (**G**) established by sh-circMMD-1 or sh-NC DBTRG cells, and survival time of mice (**H**). Scale bar = 1000 μm or 100 μm. * *P* < 0.05, ** *P* < 0.01, *** *P* < 0.001, # *P* > 0.05

Then we explored the function of circMMD in GBM cells. Three shRNAs were designed targeting the back-spliced junction of circMMD, in which sh-1 efficiently knocked down circMMD in DBTRG and U251 cells, and circMMD overexpression (OE) successfully increased circMMD expression (Fig. S1). The knockdown of circMMD significantly repressed the proliferation of DBTRG and U251 cells and also enhanced TTF-mediated suppression of cell proliferation, whereas its overexpression exerted opposite effects (Fig. 2A, B). Similarly, the percentage of EdU-positive cells was reduced by the knockdown of circMMD and further reduced by TTF, whereas the overexpression of circMMD could promote the percentage of EdU-positive cells and partially reverse TTF-mediated suppression of cell proliferation (Fig. 2C). Moreover, the manipulation of circMMD in DBTRG and U251 cells did not affect its intrinsic apoptosis, but with TTF intervention, the knockdown of circMMD could enhance TTF-mediated cell apoptosis, and its overexpression had reversed effects (Fig. 2D). To verify the function of circMMD in vivo, we established subcutaneous and intracranial orthotopic tumor model. The knockdown of circMMD significantly reduced GBM tumor volume, and Ki67 expression was lower in sh-1 group compared with sh-NC group (Fig. 2E, F). In orthotopic tumor model, the knockdown of circMMD markedly reduced GBM tumor growth and prolonged mice survival (Fig. 2G, H). Therefore, these results highlighted the oncogenic role of circMMD in GBM and its potential to be a target of TTF to mediate therapeutic effects.

### TTF impeded the binding of EIF4A3 with flank region of circMMD

**Fig. 3:**
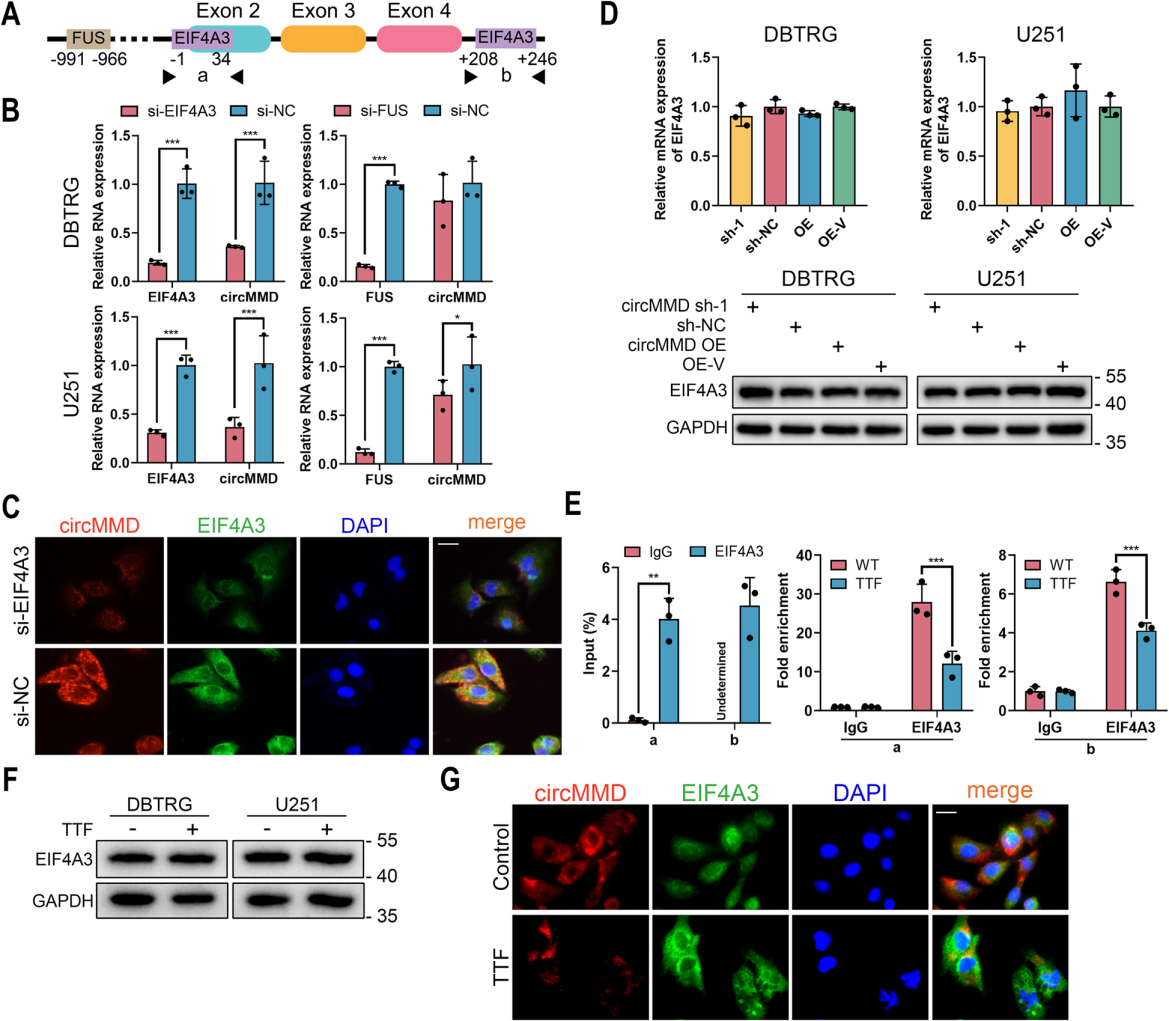
TTF impeded the binding of EIF4A3 with flank region of circMMD to inhibit its synthesis. **A** Predicted binding sites of EIF4A3 and FUS with the flank region of circMMD by CircInteractome. **B** Relative expression of EIF4A3 or FUS and circMMD after knockdown of EIF4A3 or FUS in DBTRG and U251 cells. **C** FISH and IF of circMMD and EIF4A3 in U251 cells after EIF4A3 knockdown. Scale bar = 20 μm. **D** The expression of EIF4A3 in DBTRG and U251 cells after knockdown or overexpression of circMMD. **E** RIP assay detected the binding of EIF4A3 with “a” and “b” regions with or without TTF intervention for 48 h. **F** The protein content of EIF4A3 in DBTRG and U251 cells after TTF intervention for 48 h. **G** FISH and IF of circMMD and EIF4A3 in U251 cells after TTF intervention for 48 h. Scale bar = 20 μm. * *P* < 0.05, ** *P* < 0.01, *** *P* < 0.001, # *P* > 0.05

Further, we were curious about the mechanism of TTF to reduce circMMD expression. The synthesis of circRNA could be promoted by the binding of RBPs such as EIF4A3 and FUS [7, 8]. Therefore, we predicted the potential RBPs of circMMD on CircInteractome [23]. EIF4A3 and FUS were predicted to bind with the flank region of circMMD (Fig. 3A). The knockdown of EIF4A3 was shown to reduce circMMD expression in DBTRG and U251 cells, in contrast, that of FUS had no consistent efficiency to modulate circMMD expression (Fig. 3B). FISH and IF assays revealed that the location of EIF4A3 and circMMD was overlapped and the reduction of EIF4A3 would result in decreased circMMD (Fig. 3C). Meanwhile, the knockdown or overexpression of circMMD would not change the expression of EIF4A3, indicating that circMMD was downstream of EIF4A3 (Fig. 3D). Further, EIF4A3 was shown to bind with the “a” (-1 ~  + 34) and “b” (+ 208 ~  + 246) of the flank region of circMMD, and TTF intervention would reduce their bindings (Fig. 3E). Also, we found that TTF intervention did not change the expression of EIF4A3 (Fig. 3F) but reduce the expression of circMMD (Fig. 3G). These results indicated that TTF impeded the binding of EIF4A3 with flank region of circMMD to reduce its expression.

### circMMD activated Wnt/β-catenin pathway and interacted with FUBP1

**Fig. 4:**
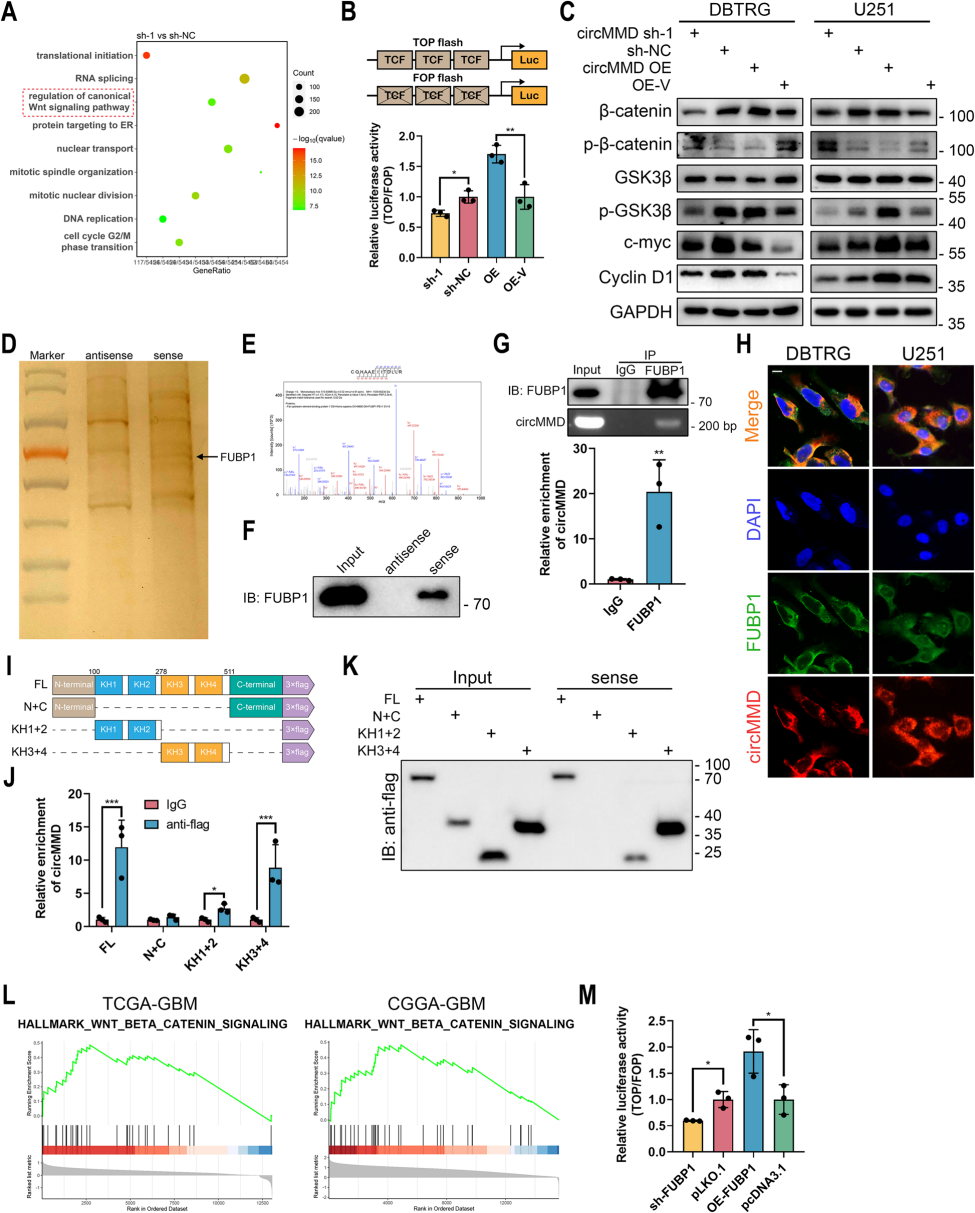
circMMD activated Wnt/β-catenin pathway and interacted with FUBP1. **A** Enrichment analysis of differentially expressed genes between sh-circMMD-1 and sh-NC DBTRG cells. **B** TOP/FOP flash assay of TCF activity affected by knockdown or overexpression of circMMD. **C** Western blot of Wnt signaling-related markers (β-catenin, GSK3β, c-myc and cyclin D1) in DBTRG and U251 cells after knockdown or overexpression of circMMD. **D** Silver staining of proteins extracted by RNA pull-down. **E–G** Mass spectrometry (**E**), western blot (**F**) and RIP (**G**) validated the interaction between circMMD with FUBP1. **H** FISH and IF detect the colocalization of circMMD and FUBP in DBTRG and U251 cells. Scale bar = 10 μm. **I** Illustration of truncated FUBP1. **J-K** RIP (**J**) and RNA pull-down (**K**) revealed KH1-4 domains of FUBP1 could bind with circMMD. **L** GSEA analysis revealed the association between FUBP1 and Wnt/β-catenin signaling in TCGA-GBM and CGGA-GBM datasets. **M** TOP/FOP flash assay of TCF activity after knockdown or overexpression of FUBP1. * *P* < 0.05, ** *P* < 0.01, *** *P* < 0.001, # *P* > 0.05

To further explore the molecular mechanism of circMMD in GBM, we conducted RNA-seq in circMMD knockdown and control cells, which identified differentially expressed genes (DEGs) between the two groups (Fig. S2A, Table S4). Enrichment analysis of DEGs revealed that circMMD might regulate canonical Wnt/β-catenin pathway and DNA replication, mitotic nuclear division, etc. (Fig. 4A), which was consistent with TTF-mediated effects as previously reported [17]. Hence, the role of circMMD in TTF was further demonstrated. TOP/FOP flash assay and western blot revealed that the knockdown of circMMD reduced the transcriptional activity of T-cell factor (TCF), whereas its overexpression could activate Wnt/β-catenin pathway (Fig. 4B, C, Fig. S3). The promoted expression of β-catenin and c-myc was also confirmed in subcutaneous xenografts (Fig. S2B). Previous studies indicated that circRNA could act as a protein scaffold to regulate protein function [5]. Therefore, we conducted RNA pull-down and mass spectrometry to identify potential binding proteins (Table S5), in which FUBP1 was selected with abundant binding peptides extracted by circMMD sense probe (Fig. 4D, E). Their interaction was further validated by western blot and RIP (Fig. 4F, G). Also, circMMD and FUBP1 were found to be colocalized (Fig. 4H). To further explore the binding site of circMMD with FUBP1, we truncated the FUBP1 into three segments based on its domains with 3 × flag tag: N-terminal and C-terminal (N + C), KH1 and KH2 (KH1 + 2), and KH3 and KH4 (KH3 + 4) (Fig. 4I). Co-IP assay confirmed the successful construction of truncated FUBP (Fig. S2C). The KH1 ~ 4 was shown to bind with circMMD and specifically, KH3 and KH4 were the main binding domains (Fig. 4J, K). Then we explored whether FUBP1 was downstream of circMMD to regulate Wnt/β-catenin pathway. The knockdown or overexpression of circMMD did not change the expression of FUBP1 (Fig. S2D-E), and the modulation of FUBP1 expression had no effect on circMMD (Fig. S2F). Moreover, through GSEA analysis, we found that FUBP1 might activate Wnt/β-catenin pathway (Fig. 4L), which was further demonstrated by TOP/FOP flash assay (Fig. 4M). Therefore, these findings demonstrated that circMMD might interact with FUBP1 to promote Wnt/β-catenin pathway.

### FUBP1 promoted DVL1 transcription by binding to its enhancer region

**Fig. 5:**
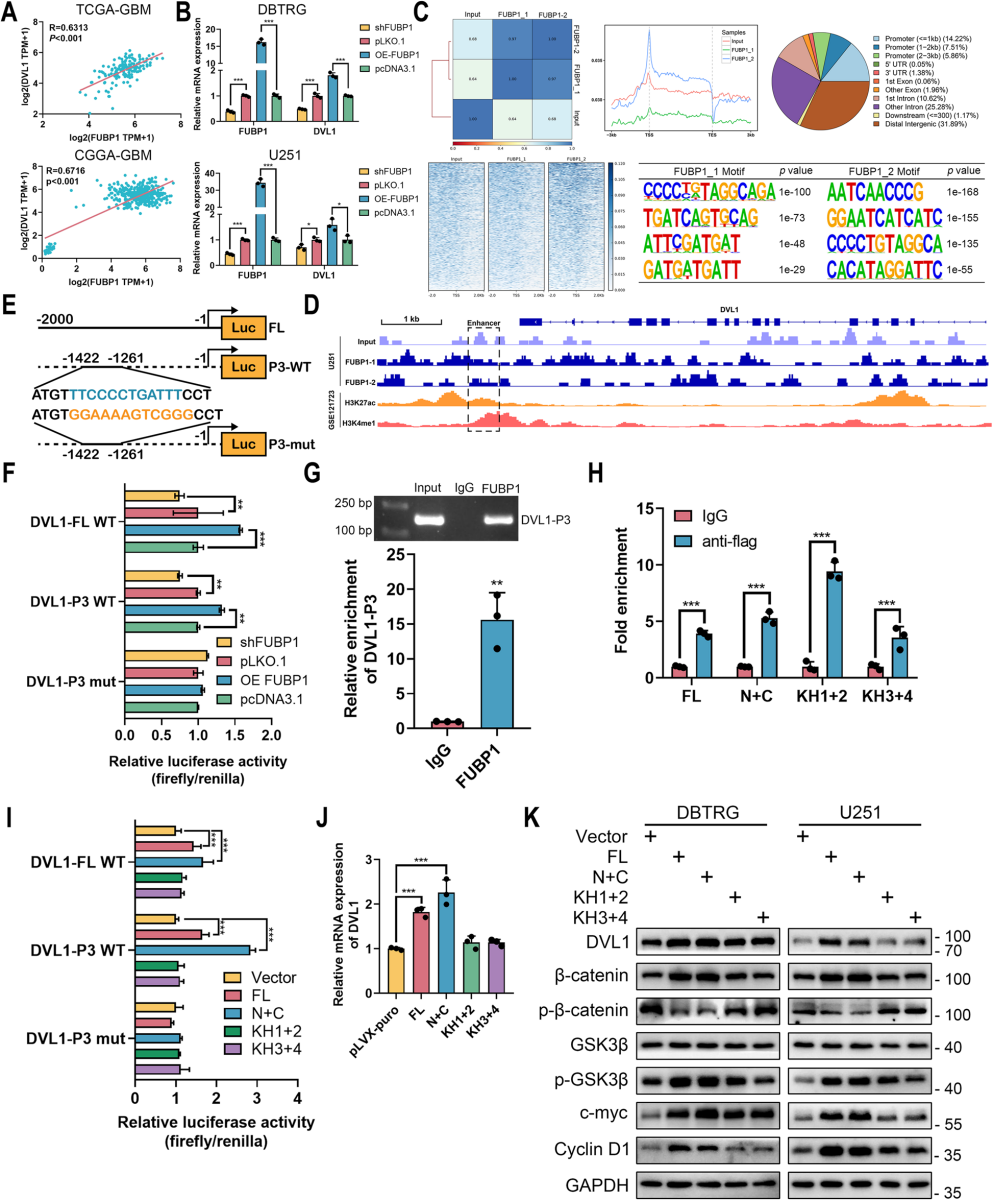
FUBP1 promoted DVL1 transcription by binding to its enhancer region. **A** Peason correlation analysis of FUBP1 and DVL1 in TCGA-GBM and CGGA-GBM datasets. **B** Relative expression of FUBP1 and DVL1 in DBTRG and U251 cells after knockdown or overexpression of FUBP1. **C** ChIP-seq of FUBP1 in U251 cells. Distribution of FUBP1-binding sequence around transcription start/end site was shown. The binding motif of FUBP1 was analyzed. **D** IGV visualization of FUBP1-binding site with DVL1 enhancer. **E** Graphic presentation of DVL1-full length (FL), DVL1-P3 wildtype and mutant luciferase vectors. **F** Dual-luciferase reporter assay detect the effect of FUBP1 on DVL1 transcription. **G-H** ChIP detected the binding domain of FUBP1 with DVL1-P3. **I** Dual-luciferase reporter assay revealed the N + C domain of FUBP1 could promote DVL1 transcription. **J** Relative expression of DVL1 in 293 T cells after transfection of different truncated FUBP1 domains. **K** The effect of different truncated FUBP1 domains on the activation of Wnt/β-catenin signaling in DBTRG and U251 cells. ** *P* < 0.01, *** *P* < 0.001, # *P* > 0.05

A previous study indicated that FUBP1 could promote DVL1 transcription to activate Wnt/β‐catenin pathway in colorectal cancer [24]. Therefore, we explored the correlation between FUBP1 and DVL1 in GBM. In TCGA-GBM and CGGA-GBM datasets, the expression of FUBP1 was positively associated with that of DVL1 (Fig. 5A). In DBTRG and U251 cells, the knockdown of FUBP1 could reduce the mRNA level of DVL1 and its overexpression could promote DVL1 expression (Fig. 5B). Meanwhile, DVL1 expression was reduced in circMMD sh-1 xenograft (Fig. S4A). Therefore, we hypothesized that FUBP1 could promote DVL1 transcription in GBM. Based on previous epigenetic profiling in GBM [21], we identified an enhancer region at downstream of DVL1 (Fig. S4B). To explore whether FUBP1 could bind to this enhancer region, we conducted ChIP-seq and revealed a binding peak of FUBP1 at the downstream enhancer of DVL1 (Fig. 5C, D). According to previous results, the “P3” segment (-1422 ~ -1261) of the 2 kb downstream of DVL1 was the binding site of FUBP1 in colorectal cancer [24]. Therefore, we constructed a luciferase vector and revealed that the knockdown of FUBP1 could reduce DVL1 transcriptional activity of FL and P3 wildtype vectors, whereas FUBP1 could promote their transcriptional activities (Fig. 5E, F). However, the effect of FUBP1 on P3 mutant vectors was not detected. Further, the binding of FUBP1 with DVL1-P3 was validated (Fig.5G), and every domain of FUBP1 was shown to bind to DVL1-P3, where the KH1 and KH2 domains were the majority (Fig. 5H). Specifically, the N + C domains of FUBP1 were responsible for transcriptional activation to promote DVL1 mRNA level (Fig. 5I, J). The promotion of DVL1 by FUBP1 and its N + C domains was shown to activate Wnt/β‐catenin pathway (Fig. 5K). These results demonstrated that FUBP1 could promote DVL1 transcription via binding to its enhancer region.

### circMMD reduced the interaction between FUBP1 and FIR to promote DVL1 transcription

**Fig. 6:**
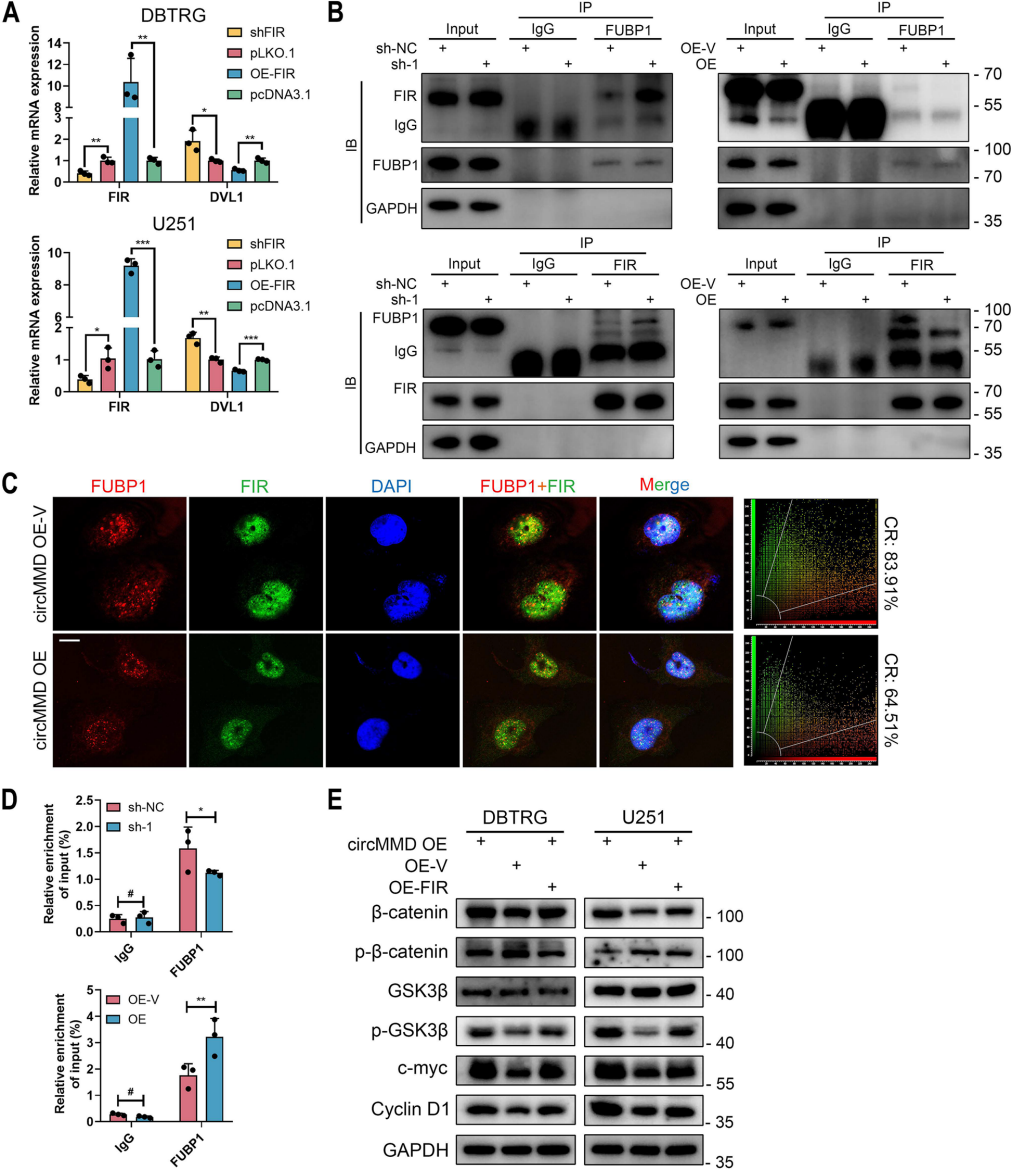
circMMD inhibited the interaction between FUBP1 and FIR to promote DVL1 transcription. **A** Relative expression of FIR and DVL1 in DBTRG and U251 cells after knockdown or overexpression of FIR. **B** Co-IP assay detect the interaction between FUBP1 and FIR in circMMD knockdown or overexpression U251 cells. **C** IF and confocal microscopy detected the colocalization of FUBP1 and FIR in circMMD knockdown or overexpression U251 cells. Scale bar = 10 μm. CR, colocalization rate. **D** ChIP assay detect the enrichment of FUBP1 at DVL1-P3 in circMMD knockdown or overexpression U251 cells. **E** Western blot of Wnt/β-catenin signaling after co-transfection of circMMD overexpression with FIR overexpression or sh-circMMD with sh-FIR in DBTRG and U251 cells. * *P* < 0.05, ** *P* < 0.01, *** *P* < 0.001, # *P* > 0.05

After we confirmed the interaction between FUBP1 and DVL1 enhancer, we investigated whether circMMD could regulate their interaction. FBP-interacting repressor (FIR) was an inhibitor of FUBP1 and was demonstrated to inhibit the interaction between FUBP1 with c-myc promoter [25]. Meanwhile, previous studies reported that long non-coding RNA (lncRNA) or circRNA could inhibit the interaction between FUBP1 and FIR to promote c-myc transcription [26, 27]. Therefore, we hypothesized that cricMMD might regulate the interaction between FUBP1 and FIR to modulate DVL1 transcription. In DBTRG and U251 cells, the knockdown of FIR could promote the expression of DVL1 whereas its overexpression reduced DVL1 expression (Fig. 6A), indicating a potential regulatory mechanism between FIR and DVL1. Further, we confirmed that FUBP1 could interact with FIR and specifically the KH1 + 2 domains were responsible for the interaction (Fig. S5). When circMMD was inhibited, the interaction between FUBP1 and FIR was promoted, and the overexpression of circMMD would inhibit their interaction (Fig. 6B). Moreover, under confocal imaging, the colocalization rate of FUBP1 and FIR was reduced after circMMD overexpression (Fig. 6C). It was known that the interaction between FUBP1 and FIR would reduce the binding of FUBP1 with DNA [25]. We found that the knockdown of circMMD would reduce the binding of FUBP1 with DVL1-P3, and its overexpression promoted their binding (Fig. 6D), which might be caused by alternated interaction between FUBP1 and FIR. Then, we demonstrated that overexpressed FIR could partially reverse circMMD-mediated activation of Wnt/β‐catenin pathway (Fig. 6E). Hence, these results revealed that circMMD could block the interaction between FUBP1 and FIR to promote DVL1 transcription, which activated Wnt/β‐catenin pathway.

### circMMD sponged miR-15b-5p to increase FZD6 expression that activated Wnt/β-catenin pathway

**Fig. 7:**
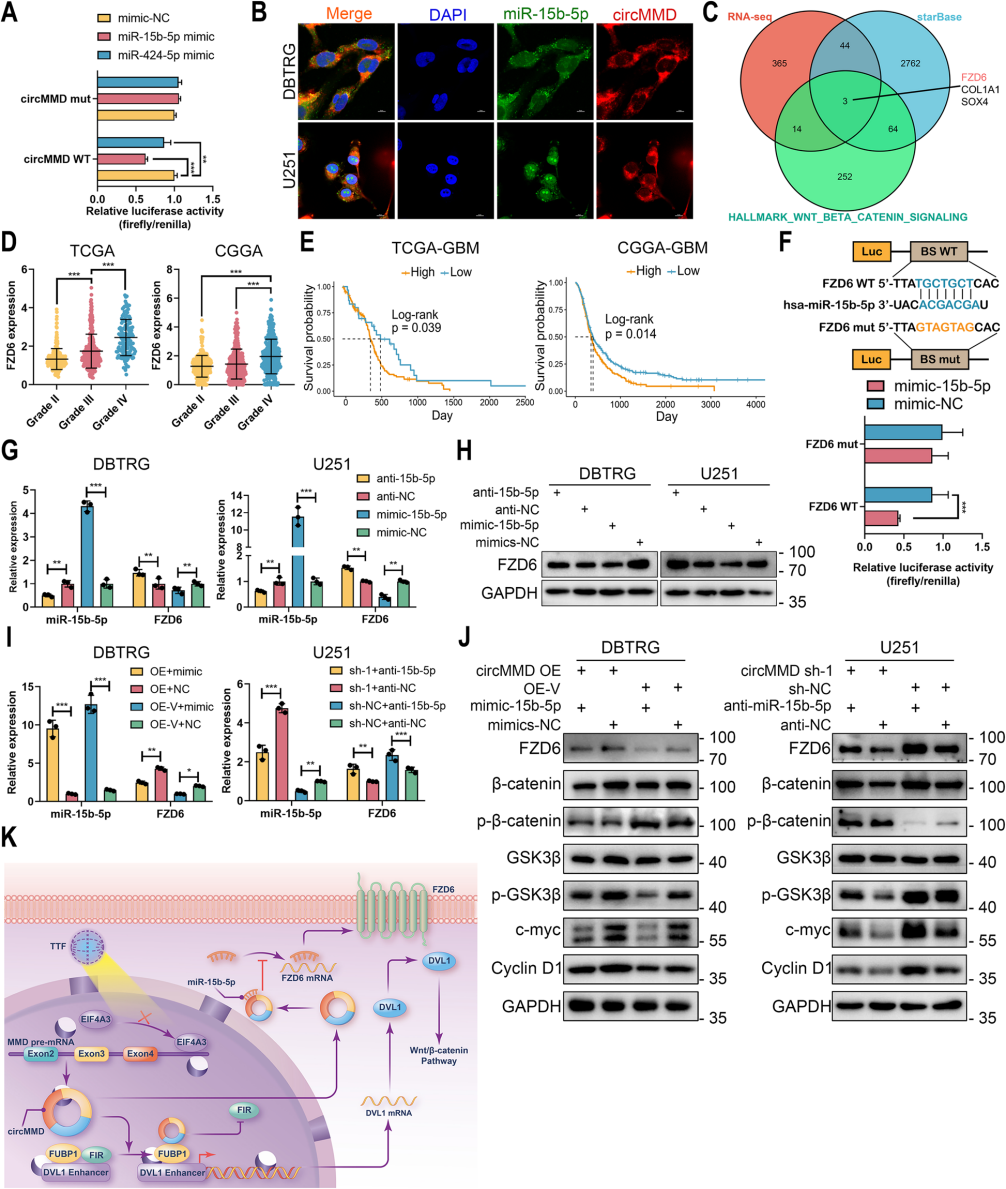
circMMD sponged miR-15b-5p to increase FZD6 expression and activated Wnt/β-catenin pathway. **A** Dual-luciferase reporter assay explored the binding of miR-15b-5p or miR-424-5p with circMMD. **B** FISH assay explore the colocalization of circMMD and miR-15b-5p. Scale bar = 10 μm. **C** FZD6 was selected among overlapped genes among RNA-seq of circMMD, starBase-predicted target genes, and gene list of Wnt/β-catenin signaling. **D** The RNA level of FZD6 in grade II, grade III, and grade IV gliomas in TCGA and CGGA datasets. **E** The prognostic value of FZD6 in GBM samples of TCGA and CGGA datasets. **F** Dual-luciferase reporter assay explored the binding of miR-15b-5p with FZD6. **G-H** The expression of miR-15b-5p and FZD6 in DBTRG (**G**) and U251 (H) cells after transfection of miR-15b-5p inhibitor or mimics. **I-J** The expression of miR-15b-5p and FZD6 (I) and the change of Wnt/β-catenin signaling (J) after co-transfection of circMMD OE with miR-15b-5p mimics or or sh-circMMD with miR-15b-5p inhibitor in DBTRG and U251 cells. **K** Schematic illustration of this study that TTF-mediated reduction of circMMD suppressed Wnt/β-catenin signaling via FUBP1/FIR/DVL1 and miR-15b-5p/FZD6 pathways. * *P* < 0.05, ** *P* < 0.01, *** *P* < 0.001, # *P* > 0.05

Among RBPs of circMMD, AGO2 was also identified, indicating a sponge of miRNA by circMMD (Table S5). Based on previous results, five miRNAs were predicted to bind with circMMD, and only two (hsa-miR-15b-5p and hsa-miR-424-5p) were differentially expressed in TTF intervention group [17]. Dual-luciferase reporter assay revealed that circMMD could bind with both miR-15b-5p and miR-424-5p, and miR-15b-5p had relatively high efficiency of binding (Fig. 7A), therefore, miR-15b-5p was selected for further analysis. FISH assay indicated colocalization of circMMD and miR-15b-5p (Fig. 7B). To screen potential target of miR-15b-5p that regulated by circMMD, we selected overlapped genes among RNA-seq result of circMMD, predicted target of miR-15b-5p at starBase, and hallmark gene set of Wnt/β-catenin pathway, in which FZD6 was selected for its essential role in transducing Wnt-signaling (Fig. 7C). In TCGA, CGGA, CGGA301, GSE108474 and GSE16011 datasets, the expression of FZD6 was elevated in a higher grade of gliomas and GBM had the highest level of FZD6 (Figs. 7D, S6A-C). Meanwhile, the high expression of FZD6 indicated poor prognosis not only in GBM but also in gliomas and LGG of TCGA, CGGA, CGGA301, GSE108474 and GSE16011 datasets (except GSE108474-GBM dataset) (Fig. 7E, S6D-H). Dual-luciferase assay confirmed the binding of miR-15b-5p with FZD6 mRNA (Fig. 7F). The transfection of miR-15b-5p inhibitor or mimics could promote or reduce FZD6 expression, respectively (Fig. 7G, H). To validate the correlation between circMMD and miR-15b-5p, we transfected the miR-15b-5p mimics in circMMD overexpressed DBTRG cells and miR-15b-5p inhibitor in circMMD knockdown U251 cells. The miR-15b-5p mimics could reduce FZD6 expression and suppress the phosphorylation of β-catenin and β-GSK3β, which partially rescued circMMD-mediated activation of Wnt/β-catenin signaling; in contrast, miR-15b-5p inhibitor could promote FZD6 expression to activate Wnt/β-catenin signaling, which was suppressed by the knockdown of circMMD (Fig. 7I, J). Therefore, circMMD could sponge miR-15b-5p to promote FZD6 expression, which activated Wnt/β-catenin pathway.

## Discussion

TTF is the latest treatment for GBM, and its molecular mechanism remains largely unknown. Based on previous transcriptomic profiling [17], we identified circMMD as a potential target of TTF. TTF interrupted the interaction between EIF4A3 and circMMD flank region to reduce the synthesis of circMMD (Fig. 7K). The knockdown of circMMD was shown to suppress GBM proliferation and growth both in vitro and in vivo. Mechanistically, circMMD could block the interaction between FUBP1 and FIR to enhance FUBP1-mediated promotion of DVL1 transcription, which activated Wnt/β-catenin pathway. Meanwhile, circMMD could sponge miR-15b-5p to increase FZD6 expression, which simultaneously activated Wnt/β-catenin pathway. Overall, circMMD may be a prognostic biomarker for GBM patients and a potential target to enhance TTF-mediated efficacy in GBM.

EIF4A3 is shown to promote the biogenesis of several circRNAs that regulate tumorigenesis in different cancers [7, 28, 29]. EIF4A3 usually binds to the downstream region of circRNAs to promote their expressions. In this study, we found that EIF4A3 could promote the synthesis of circMMD but its expression was not changed by TTF intervention. Since proteins and RNAs had electric charges, we hypothesized that TTF-induced electric field might affect the binding of EIF4A3 with circMMD flank region, which was further demonstrated by RIP assay. This finding uncovered a novel mechanism that TTF regulated the expression of circRNA.

FUBP1 is a multifunctional DNA and RNA binding protein. It can bind to the FUSE element on the promoter of *MYC* to promote its transcription, which is suppressed by FIR [30, 31]. This dynamic regulation exists in cells to maintain the stable expression of oncogene *MYC*. However, in tumor cells, the interaction between FUBP1 and FIR could be affected by other factors such as lncRNA or circRNA, which resulted in increased expression of c-myc and promoted tumorigenesis [26, 27]. Meanwhile, FUBP1 was shown to promote DVL1 transcription in colorectal cancer [24]. Therefore, we hypothesized that circMMD might affect the interaction between FUBP1 and FIR to regulate DVL1 transcription. In this study, we demonstrated that KH1-4 domains of FUBP1 were responsible for binding with circMMD, KH1 and KH2 domains were responsible for FIR binding, all domains of FUBP1 could bind to DVL1 enhancer, and N + C domains could promote DVL1 transcription. Liu et al. revealed that the C-terminal of FUBP1 was the transactivation domain that could bind to the p89 subunit of transcription factor IIH (TFIIH) [32], which accounted for our results that N + C domains of FUBP1 were able to enhance DVL1 transcription. Meanwhile, it appeared strange that N + C domains could bind to DVL1 enhancer. This might be due to the cross-link of C-terminal domain with TFIIH, which directly contacted to DVL1 enhancer, by formaldehyde at the first step of ChIP. When we pull down N + C domains using anti-flag antibody, TFIIH was also extracted together with DVL1 enhancer, which was detected by qPCR. Additionally, our study demonstrated that circMMD could reduce the interaction between FUBP1 and FIR to promote DVL1 transcription, which revealed a novel mechanism to regulate Wnt/β-catenin pathway.

The most common function of circRNA is to serve as miRNA sponge. The first circRNA identified as the competing endogenous RNA was CDR1as, which could bind to miR-7 with more than 60 conserved binding sites [33]. The ceRNA network in TTF intervention was screened in our previous study [17], which indicated that circMMD could bind to several miRNAs. Among these miRNAs, miR-15b-5p was identified as the target of circMMD. Moreover, among three overlapped candidate target genes of miR-15b-5p, FZD6 was selected due to its essential role in Wnt/β-catenin pathway and its prognostic role in GBM. FZD6 is a 7-transmembrane domain protein serving as the receptor of Wnt signaling. After the interaction between Wnt and FZD, DVL would be recruited and activated to suppress the formation of a degradation complex composed of GSK3β, APC, AXIN, and CK1, which inhibited the degradation of β-catenin and stabilized its translocation into the nucleus [34]. Our findings revealed that circMMD could not only promote FZD6 expression but also increase DVL1 transcription, which resulted in the activation of Wnt/β-catenin pathway.

To sum up, our study revealed that TTF could decrease EIF4A3-mediated biogenesis of circMMD, which suppressed the activation of Wnt/β-catenin pathway and inhibited GBM proliferation. circMMD could be a prognostic biomarker for GBM patients and a therapeutic target to enhance TTF efficacy.

## Supplementary Information

**Additional file 1: Figure S1.** Effect of three shRNAs targeting circMMD and overexpression plasmid on circMMD expression in DBTRG and U251 cells. **Figure S2.** Association between circMMD with Wnt/β-catenin pathway and FUBP1. **Figure S3.** Nuclear and cytoplasmic content of β-catenin after knockdown of circMMD. **Figure S4.** FUBP1 could bind to DVL1 enhancer region. **Figure S5.** Interaction between truncated FUBP with FIR. **Figure S6.** Expression pattern and prognostic value of FZD6 in glioma datasets.

**Additional file 2: Table S1.** Information of the primers, siRNAs/shRNAs and antibodies used in the study.

**Additional file 3: Table S2.** Clinical information of glioma array.

**Additional file 4: Table S3.** Correlation between circMMD expression and clinical features of glioma patients.

**Additional file 5: Table S4.** Differentially expressed genes between circMMD knockdown and NC groups.

**Additional file 6: Table S5.** Candidate proteins extracted by circMMD sense probe.

## Data Availability

All data generated or analyzed during this study are included in this published article and its supplementary information files.

## Abbreviations

GBM: Glioblastoma
OS: Overall survival
TTF: Tumor treating fields
circRNA: Circular RNA
EIF4A3: Eukaryotic translation initiation factor 4A3
FUS: FUS RNA binding protein
TCGA: The Cancer Genome Atlas
CGGA: Chinese Glioma Genome Atlas
GEO: Gene Expression Omnibus
LGG: Lower-grade glioma
ChIP: Chromatin immunoprecipitation
IGV: Integrative Genomics Viewer
EdU: 5-Ethynyl-2’-Deoxyuridine
IHC: Immunohistochemistry
HE: Hematoxylin–eosin
DAB: Diaminobenzidine tetrahydrochloride
RIP: RNA immunoprecipitation
GO: Gene Ontology
KEGG: Kyoto Encyclopedia of Genes and Genomes
BP: Biological process
CC: Cellular component
MF: Molecular function
FDR: False discovery rate
Co-IP: Co-immunoprecipitation
DEGs: Differentially expressed genes
TCF: T-cell factor
FIR: FBP-interacting repressor
lncRNA: Long non-coding RNA
TFIIH: Transcription factor IIH
