# Supplementary material for: circMMD reduction following tumor treating fields inhibits glioblastoma progression through FUBP1/FIR/DVL1 and miR-15b-5p/FZD6 signaling
Source: J Exp Clin Cancer Res. 2023 Mar 17;42:64. doi: 10.1186/s13046-023-02642-z (PMC10021944; doi:10.1186/s13046-023-02642-z)
Supplement: Supplementary file 1 — Additional file 1: Figure S1. Effect of three shRNAs targeting circMMD and overexpression plasmid on circMMD expression in DBTRG and U251 cells. Figure S2. Association between circMMD with Wnt/β-catenin pathway and FUBP1. Figure S3. Nuclear and cytoplasmic content of β-catenin after knockdown of circMMD. Figure S4. FUBP1 could bind to DVL1 enhancer region. Figure S5. Interaction between truncated FUBP with FIR. Figure S6. Expression pattern and prognostic value of FZD6 in glioma datasets. [file 13046_2023_2642_MOESM1_ESM.docx]

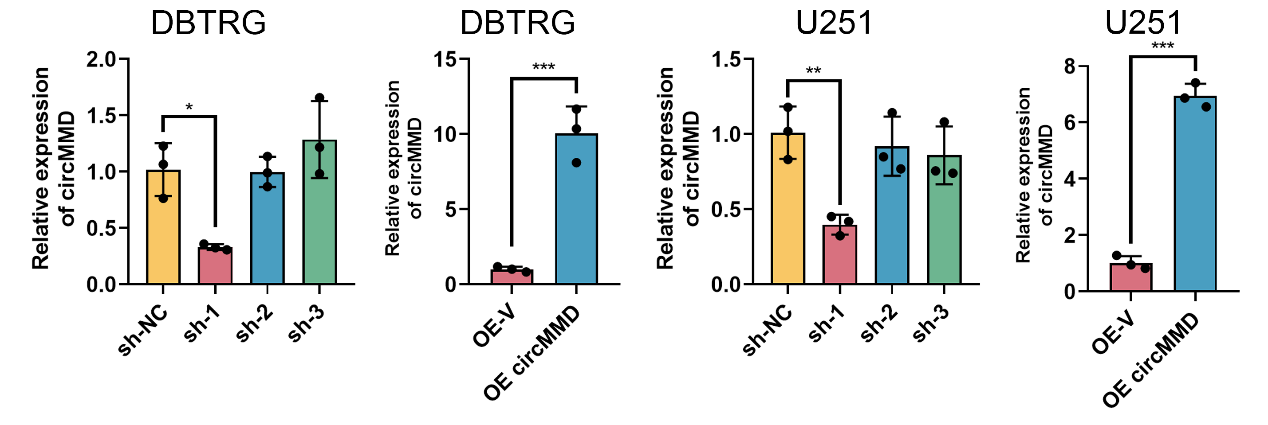


**Figure S1. Effect of three shRNAs targeting circMMD and overexpression plasmid on circMMD expression in DBTRG and U251 cells.**

**
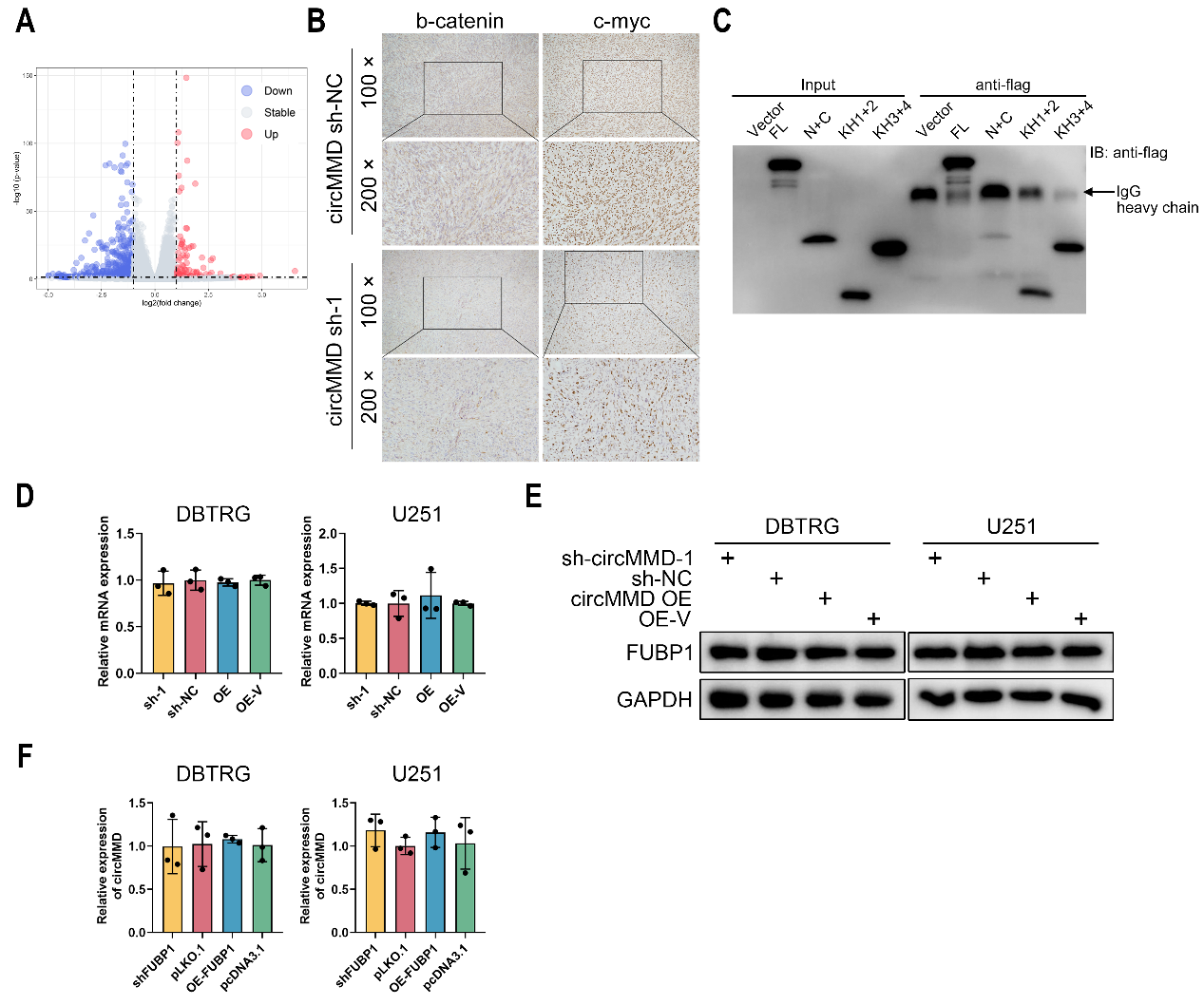
**

**Figure S2. Association between circMMD with Wnt/β-catenin pathway and FUBP1. A.** Volcano plot of differentially expressed genes between circMMD knockdown and control groups. **B.** IHC detected β-catenin and c-myc in circMMD knockdown and control xenografts. **C.** Co-IP confirmed the successful construction of truncated FUBP1 domains. **D-E.** The expression of FUBP1 in DBTRG and U251 cells after circMMD knockdown or overexpression. **F.** The expression of circMMD in DBTRG and U251 cells after FUBP1 knockdown or overexpression.


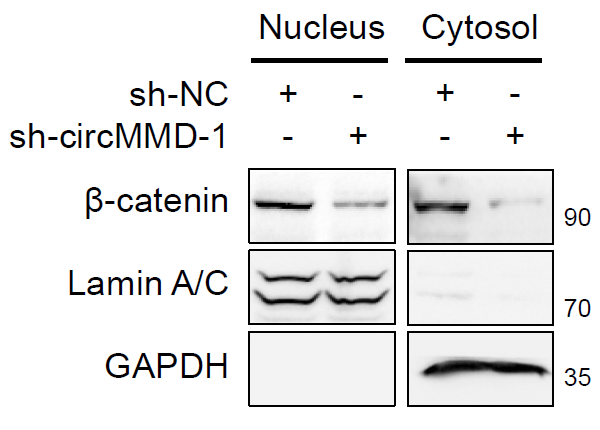


**Figure S3. Nuclear and cytoplasmic content of β-catenin after knockdown of circMMD.**


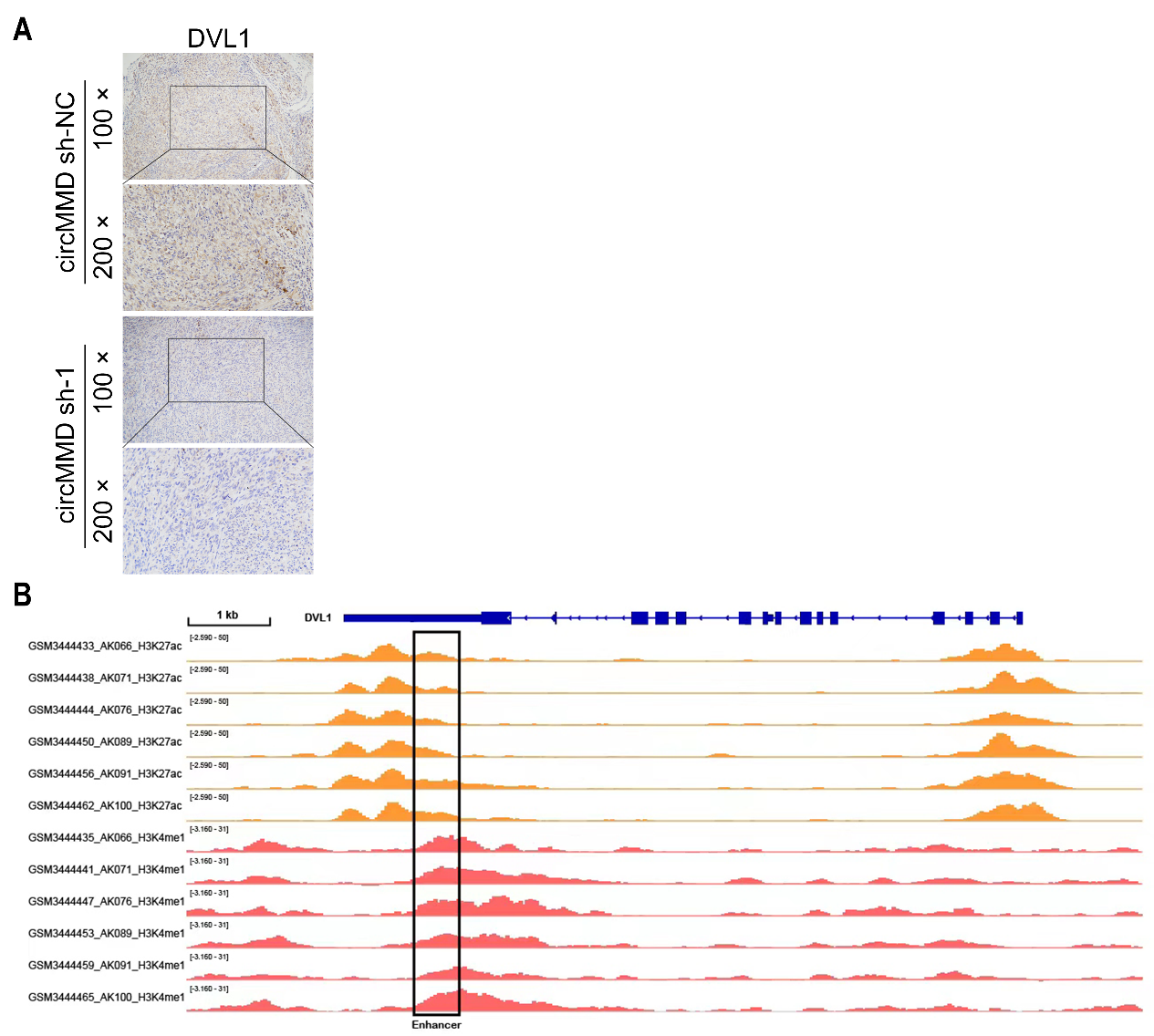


**Figure S4. FUBP1 could bind to DVL1 enhancer region. A.** IHC detected DVL1 expression in circMMD knockdown and control xenografts. **B.** The enrichment of H3K27ac and H3K4me at downstream of DVL1 indicated an enhancer region.


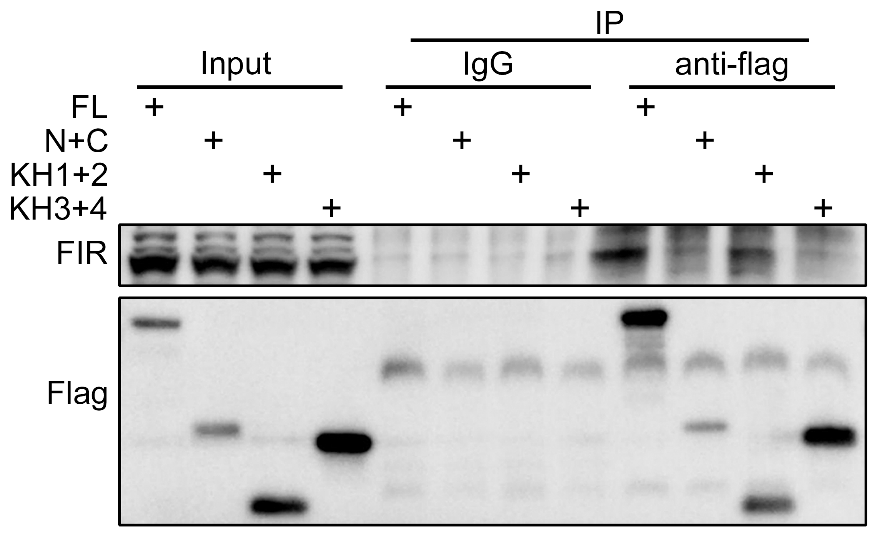


**Figure S5. Interaction between truncated FUBP with FIR.**


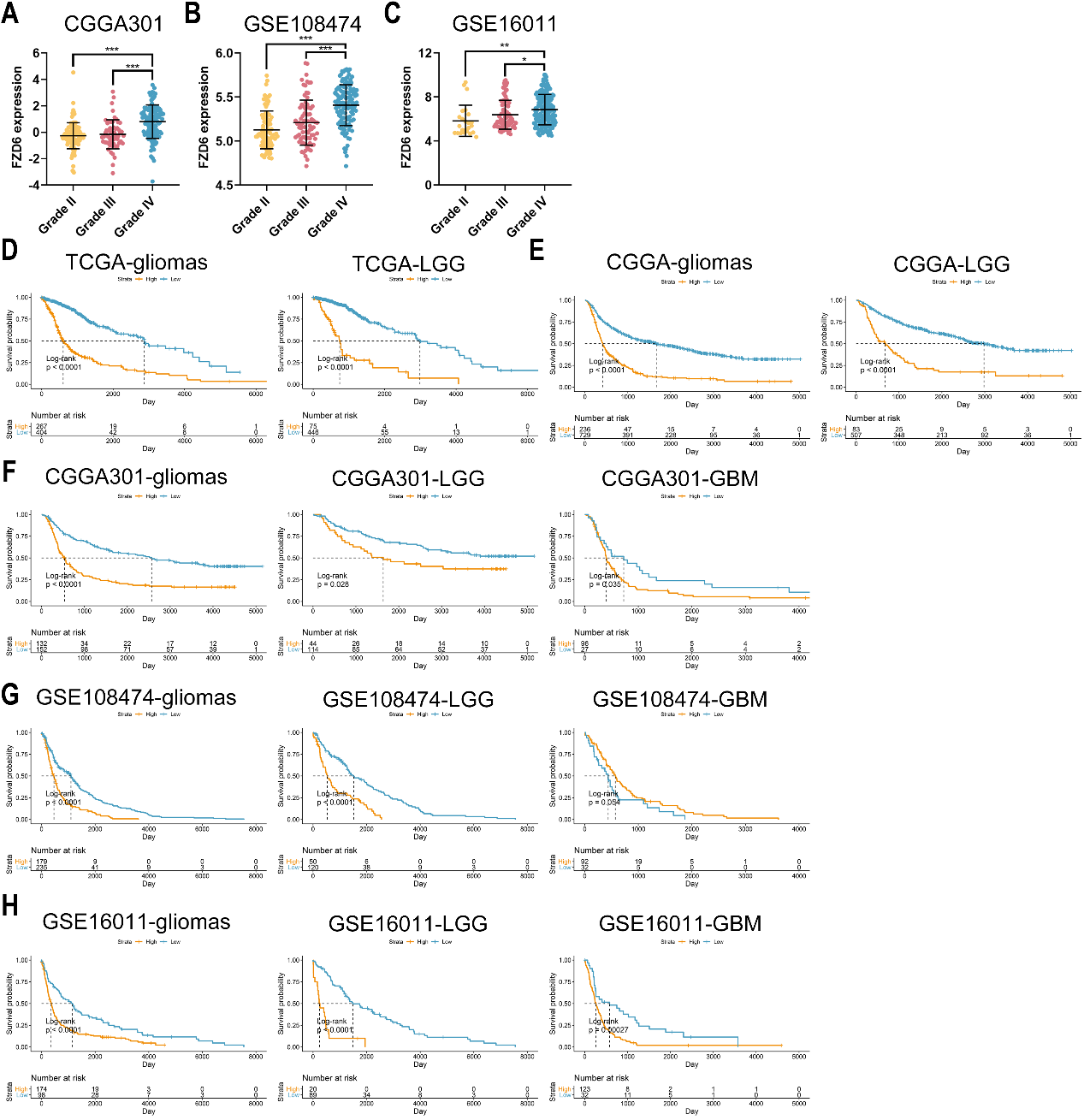


**Figure S6. Expression pattern and prognostic value of FZD6 in glioma datasets. A-C.** The RNA level of FZD6 in grade II, grade III, and grade IV gliomas in CGGA301 (A), GSE108474 (B), and GSE16011 (C) datasets. **D-E.** The prognostic value of FZD6 in gliomas and LGG in TCGA (D) and CGGA (E) datasets. **F-H.** The prognostic value of FZD6 in gliomas, LGG and GBM samples in CGGA301 (F), GSE108474 (G), and GSE16011 (H) datasets. * *P* < 0.05, ** *P* < 0.01, *** *P* <0.001.
